# 1,4-Bis(4-bromo-2,6-diisopropyl­phen­yl)-1,4-diaza­buta-1,3-diene

**DOI:** 10.1107/S1600536809050843

**Published:** 2009-12-04

**Authors:** Ilia A. Guzei, Nicholas J. Hill, Matthew R. Van Hout

**Affiliations:** a Department of Chemistry, University of Wisconsin-Madison, 1101 University Ave, Madison, WI 53706, USA

## Abstract

The molecule of the title compound, C_26_H_34_Br_2_N_2_, lies on a crystallographic inversion center and hence the two imine groups are *s-trans*. The dihedral angle between the central 1,4-diaza­buta-1,3-diene unit and the attached substituted phenyl ring is 88.4 (7)°. The structure features a C—H⋯N close contact. The crystal selected for this study proved to be a non-merohedral twin with a minor component of 21.8 (3)%.

## Related literature

1,4-diaza-1,3-butadiene (DAB) ligands containing sterically demanding *N*-substituents have proved to be versatile platforms for stabilizing *s*- and *p*-block atoms in unusual oxidation states or coordination geometries, see: Baker et al. (2008 ▶); Hill et al. (2009 ▶); Liu et al. (2009 ▶); Martin et al. (2009 ▶); Segawa et al. (2008 ▶). The title compound was prepared as part of our continuing studies on the chemistry of *N*-heterocyclic silylenes and germylenes, see: Hill et al. (2005 ▶); Naka et al. (2004 ▶); Tomasik et al. (2009 ▶). For the use of DAB ligands in olefin polymerization catalysis, see: Ittel et al. (2000 ▶); Jung et al. (2007 ▶). For related structures, see: (2003); Müller et al. (2003 ▶); Schaub et al. (2006 ▶); Berger et al. (2001 ▶); Laine et al. (1999 ▶). For the preparation of 4-bromo-2,6-di-iso-propyl aniline, see: Liu et al. (2005 ▶). For a description of the Cambridge Structural Database, see: Allen (2002 ▶).
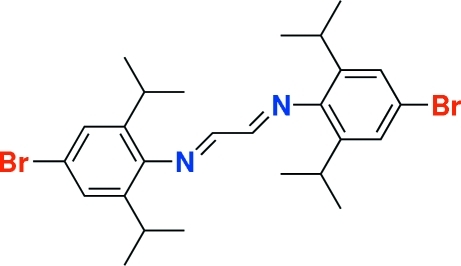


## Experimental

### 

#### Crystal data

C_26_H_34_Br_2_N_2_
*M* *_r_* = 534.37
Monoclinic, 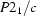

*a* = 8.961 (3) Å
*b* = 17.848 (7) Å
*c* = 8.620 (3) Å
β = 104.260 (11)°
*V* = 1336.2 (8) Å^3^
*Z* = 2
Mo *K*α radiation
μ = 3.05 mm^−1^
*T* = 300 K
0.43 × 0.35 × 0.29 mm

#### Data collection

Bruker SMART X2S diffractometer
Absorption correction: multi-scan (TWINABS; Bruker, 2007 ▶) *T*_min_ = 0.103, *T*_max_ = 0.428
2286 measured reflections
2286 independent reflections
1585 reflections with *I* > 2σ(*I*)
*R*_int_ = 0.110

#### Refinement

*R*[*F* ^2^ > 2σ(*F* ^2^)] = 0.069
*wR*(*F* ^2^) = 0.199
*S* = 1.04
2286 reflections
142 parameters
H-atom parameters constrained
Δρ_max_ = 0.53 e Å^−3^
Δρ_min_ = −0.60 e Å^−3^

### 

Data collection: *GIS* (Bruker, 2009 ▶); cell refinement: *SAINT* (Bruker, 2007 ▶); data reduction: *SAINT*; program(s) used to solve structure: *SHELXTL* (Sheldrick, 2008 ▶); program(s) used to refine structure: *SHELXTL* and *OLEX2* (Dolomanov *et al.*, 2009 ▶); mol­ecular graphics: *SHELXTL* and *OLEX2*; software used to prepare material for publication: *SHELXTL*, *OLEX2* (Dolomanov *et al.*, 2009 ▶), *publCIF* (Westrip, 2009 ▶) and *modiCIFer* (Guzei, 2007 ▶).

## Supplementary Material

Crystal structure: contains datablocks global, I. DOI: 10.1107/S1600536809050843/bx2248sup1.cif

Structure factors: contains datablocks I. DOI: 10.1107/S1600536809050843/bx2248Isup2.hkl

Additional supplementary materials:  crystallographic information; 3D view; checkCIF report

## Figures and Tables

**Table 1:** Hydrogen-bond geometry (Å, °)

*D*—H⋯*A*	*D*—H	H⋯*A*	*D*⋯*A*	*D*—H⋯*A*
C8—H8⋯N1	0.98	2.40	2.880 (9)	109

**Fig. 1.**
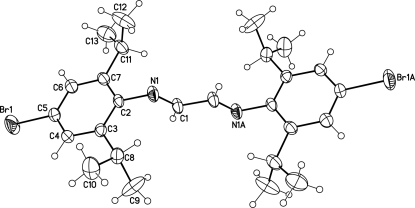
Molecular structure of (I). The thermal ellipsoids are shown at 30% probability level. Atoms labeled with the suffixes A and unlabeled are generated by the symmetry operation (-x+1, -y+1, -z+1).

## supplementary crystallographic information

### Comment

1,4-diaza-1,3-butadiene (DAB) ligands bearing bulky aryl or alkyl groups on the nitrogen atoms have proven to be versatile platforms for stabilizing s- and *p*-block atoms in unusual oxidation states or coordination geometries (Baker *et al.* (2008); Hill *et al.* (2009); Liu *et al.* (2009); Martin *et al.* (2009); Segawa *et al.* (2008)). The title compound, (I), was prepared as part of our continuing studies upon the chemistry of N-heterocyclic silylenes and germylenes (Hill *et al.* (2005); Naka *et al.* (2004); Tomasik *et al.* (2009)), the silicon(II) and germanium(II) analogues of the well known Arduengo N-heterocyclic carbenes. DAB ligands are ideal in this regard since their stereo-electronic properties are easily tuned by alteration of the N– and C-substituents.

DAB ligands have also been used extensively in d-block coordination chemistry, particularly within the field of olefin polymerization catalysis (Ittel *et al*. 2000). Jung *et al.* (2007) recently used the title compound as a precursor to an N-heterocyclic carbene in the synthesis of a catalytically active cationic (η^3^-allyl)(NHC)palladium complex.

The molecule of (I) resides on a crystallographic inversion center and hence the two imine groups are *s-trans. *The dihedral angle between the central 1,4-diazabuta-1,3-diene moiety and the attached substituted phenyl ring is 88.4 (7)°. The molecular symmetry approaches C_2h_, however, the positions of the isopropyl groups break the mirror plane symmetry: both H atoms on the tertiary C atoms of the two symmetry-independent ^i^Pr groups point toward atom N1, but reside on the opposite sides of the phenyl ring. This is illustrated with two disparate but "would be equivalent" torsion angles, one for each ^i^Pr group: C2—C3—C8—C9 (-96.5 (8)°) and C2—C7—C11—C13 (163.6 (7)°). This geometry differs from that of the unbrominated congener of (I), 1,4-bis(2,6-diisopropyl-phenyl)-1,4-diazabuta-1,3-diene, (II). For related structures, see: Müller *et al.* (2003), Schaub *et al.* (2006). Compound (II), structurally characterized at 173 K by Berger *et al.* (2001) and at 193 K by Laine *et al.*(1999), crystallizes with the molecule of (II) on an inversion center. The H atoms of the tertiary C atom of the isopropyl groups point toward the N atom and, in contrast to (I), are located on the same side of the phenyl ring. The overall symmetry of (II) is much closer to C_2h_ as the ^i^Pr groups are oriented very similarly: in the 193 K structure of (II) two "would be equivalent" Me—C(H)—C—C(N) torsion angles measured 144.6 and 145.4°. The C—Br distance of 1.897 (6) Å is in excellent agreement with the value of 1.899 (11) Å obtained by averaging 2303 C—Br bond lengths from 1736 relevant compounds reported to the Cambridge Structural Database (Allen, 2002).

### Experimental

4-Bromo-2,6-di-*iso*-propyl aniline was prepared according to the literature procedure (Liu *et al.* 2005). To a stirred solution of 4-bromo-2,6-di-*iso*-propyl aniline (3.0 g,11.71 mmol) in methanol (40 cm^3^) containing 4 drops of formic acid was added glyoxal (0.85 g, 5.80 mmol, 40% aqueous soln.) slowly dropwise. The reaction mixture was stirred for 24 h at room temperature, filtered, and the precipitate washed with cold MeOH (2 *x* 10 mL). This yellow solid was dried *in vacuo* and recrystallized from EtOH to give a crop of pale yellow needles suitable for X-ray diffraction analysis. Yield 3.53 g, 56%.

^1^H-NMR (CD_2_Cl_2_, 300 MHz): δ 1.19 (d, ^3^*J* = 6.9 Hz, 24H, CH_3_), 2.91 (sept, ^3^*J* = 6.8 Hz, 4H, CH), 7.31 (s, 4H, aromatic), 8.07 (s, 2H, CH); ^13^C{^1^H}-NMR (CD_2_Cl_2_, 75 MHz): δ 22.80, 28.43, 119.42, 126.79, 139.29, 147.52, 163.97.

### Refinement

All H-atoms were placed in idealized locations with C—H distances 0.93 - 0.98 Å and refined as riding with appropriate thermal displacement coefficients *U*_iso_(H) = 1.2 or 1.5 times *U*_eq_(bearing atom). The crystal of (I) selected for this study proved to be a non-merohedral twin. The two twin components are related by a 179.9° rotation about the [001] direction in reciprocal space with the minor component contribution of 21.8 (3)%.

### Crystal data

**Table:**

C_26_H_34_Br_2_N_2_	*F*(000) = 548
*M**_r_* = 534.37	*D*_x_ = 1.328 Mg m^−^^3^
Monoclinic, *P*2_1_/*c*	Mo *K*α radiation, λ = 0.71073 Å
Hall symbol: -P 2ybc	Cell parameters from 999 reflections
*a* = 8.961 (3) Å	θ = 2.3–24.8°
*b* = 17.848 (7) Å	µ = 3.05 mm^−^^1^
*c* = 8.620 (3) Å	*T* = 300 K
β = 104.260 (11)°	Block, yellow
*V* = 1336.2 (8) Å^3^	0.43 × 0.35 × 0.29 mm
*Z* = 2	

### Data collection

**Table:**

Bruker SMART X2S diffractometer	2286 independent reflections
Radiation source: micro-focus sealed tube	1585 reflections with *I* > 2σ(*I*)
doubly curved silicon crystal	*R*_int_ = 0.110
ω scans	θ_max_ = 24.8°, θ_min_ = 2.3°
Absorption correction: multi-scan (TWINABS; Bruker, 2007)	*h* = 0→10
*T*_min_ = 0.103, *T*_max_ = 0.428	*k* = −21→0
2286 measured reflections	*l* = −10→9

### Refinement

**Table:**

Refinement on *F*^2^	Secondary atom site location: difference Fourier map
Least-squares matrix: full	Hydrogen site location: inferred from neighbouring sites
*R*[*F*^2^ > 2σ(*F*^2^)] = 0.069	H-atom parameters constrained
*wR*(*F*^2^) = 0.199	*w* = 1/[σ^2^(*F*_o_^2^) + (0.0949*P*)^2^ + 1.843*P*] where *P* = (*F*_o_^2^ + 2*F*_c_^2^)/3
*S* = 1.04	(Δ/σ)_max_ = 0.001
2286 reflections	Δρ_max_ = 0.53 e Å^−^^3^
142 parameters	Δρ_min_ = −0.60 e Å^−^^3^
0 restraints	Extinction correction: *SHELXTL* (Sheldrick, 2008), Fc^*^=kFc[1+0.001xFc^2^λ^3^/sin(2θ)]^-1/4^
Primary atom site location: structure-invariant direct methods	Extinction coefficient: 0.038 (5)

### Special details

**Table:**

Geometry. All e.s.d.'s (except the e.s.d. in the dihedral angle between two l.s. planes) are estimated using the full covariance matrix. The cell e.s.d.'s are taken into account individually in the estimation of e.s.d.'s in distances, angles and torsion angles; correlations between e.s.d.'s in cell parameters are only used when they are defined by crystal symmetry. An approximate (isotropic) treatment of cell e.s.d.'s is used for estimating e.s.d.'s involving l.s. planes.
Refinement. Refinement of *F*^2^ against ALL reflections. The weighted *R*-factor *wR* and goodness of fit *S* are based on *F*^2^, conventional *R*-factors *R* are based on *F*, with *F* set to zero for negative *F*^2^. The threshold expression of *F*^2^ > σ(*F*^2^) is used only for calculating *R*-factors(gt) *etc*. and is not relevant to the choice of reflections for refinement. *R*-factors based on *F*^2^ are statistically about twice as large as those based on *F*, and *R*- factors based on ALL data will be even larger.

### Fractional atomic coordinates and isotropic or equivalent isotropic displacement parameters (Å^2^)

**Table:**

	*x*	*y*	*z*	*U*_iso_*/*U*_eq_	
Br1	1.14981 (9)	0.69711 (6)	0.27272 (12)	0.0957 (6)	
N1	0.6097 (6)	0.5812 (3)	0.5079 (7)	0.0523 (14)	
C1	0.5657 (8)	0.5148 (3)	0.4744 (8)	0.0513 (17)	
H1	0.6173	0.4847	0.4166	0.062*	
C2	0.7381 (6)	0.6090 (3)	0.4544 (7)	0.0395 (14)	
C3	0.7094 (7)	0.6469 (3)	0.3074 (7)	0.0409 (14)	
C4	0.8336 (7)	0.6738 (3)	0.2564 (7)	0.0456 (15)	
H4	0.8179	0.6992	0.1595	0.055*	
C5	0.9804 (7)	0.6630 (4)	0.3489 (8)	0.0500 (16)	
C6	1.0083 (7)	0.6289 (4)	0.4968 (8)	0.0529 (16)	
H6	1.1086	0.6245	0.5591	0.063*	
C7	0.8860 (8)	0.6012 (3)	0.5528 (8)	0.0485 (16)	
C8	0.5477 (7)	0.6575 (4)	0.2044 (9)	0.0536 (16)	
H8	0.4780	0.6500	0.2746	0.064*	
C9	0.5079 (12)	0.5984 (6)	0.0770 (14)	0.119 (4)	
H9C	0.5672	0.6062	−0.0004	0.179*	
H9B	0.4002	0.6013	0.0251	0.179*	
H9A	0.5309	0.5498	0.1247	0.179*	
C10	0.5185 (9)	0.7356 (5)	0.1375 (14)	0.088 (3)	
H10C	0.5496	0.7714	0.2224	0.132*	
H10A	0.4108	0.7416	0.0883	0.132*	
H10B	0.5766	0.7436	0.0590	0.132*	
C11	0.9157 (9)	0.5652 (4)	0.7203 (8)	0.0626 (18)	
H11	0.8533	0.5195	0.7090	0.075*	
C12	0.8601 (13)	0.6152 (5)	0.8316 (10)	0.101 (3)	
H12B	0.8573	0.5880	0.9269	0.151*	
H12A	0.7586	0.6327	0.7808	0.151*	
H12C	0.9285	0.6572	0.8591	0.151*	
C13	1.0834 (11)	0.5415 (5)	0.7895 (10)	0.087 (3)	
H13A	1.1158	0.5093	0.7148	0.131*	
H13B	1.0915	0.5152	0.8884	0.131*	
H13C	1.1478	0.5852	0.8086	0.131*	

### Atomic displacement parameters (Å^2^)

**Table:**

	*U*^11^	*U*^22^	*U*^33^	*U*^12^	*U*^13^	*U*^23^
Br1	0.0555 (5)	0.1428 (10)	0.0961 (8)	−0.0228 (5)	0.0324 (5)	0.0295 (6)
N1	0.067 (3)	0.034 (3)	0.065 (4)	−0.011 (2)	0.036 (3)	−0.001 (2)
C1	0.068 (4)	0.035 (3)	0.063 (4)	−0.014 (3)	0.040 (4)	−0.007 (3)
C2	0.053 (3)	0.027 (3)	0.046 (4)	−0.006 (2)	0.026 (3)	−0.004 (3)
C3	0.046 (3)	0.033 (3)	0.046 (4)	−0.005 (3)	0.015 (3)	−0.003 (3)
C4	0.053 (4)	0.046 (3)	0.039 (3)	−0.006 (3)	0.014 (3)	0.002 (3)
C5	0.046 (4)	0.059 (4)	0.050 (4)	−0.011 (3)	0.021 (3)	0.000 (3)
C6	0.052 (3)	0.057 (4)	0.050 (4)	−0.006 (3)	0.014 (3)	0.006 (3)
C7	0.064 (4)	0.042 (3)	0.043 (4)	−0.006 (3)	0.020 (3)	0.002 (3)
C8	0.046 (3)	0.052 (4)	0.063 (4)	−0.005 (3)	0.014 (3)	0.007 (3)
C9	0.093 (7)	0.119 (8)	0.117 (9)	−0.024 (6)	−0.030 (6)	−0.045 (7)
C10	0.063 (5)	0.080 (5)	0.115 (8)	0.008 (4)	0.013 (6)	0.039 (6)
C11	0.083 (5)	0.059 (4)	0.052 (4)	−0.003 (4)	0.027 (4)	0.014 (4)
C12	0.161 (10)	0.094 (6)	0.063 (6)	0.043 (6)	0.058 (6)	0.026 (5)
C13	0.109 (7)	0.081 (6)	0.072 (6)	0.025 (5)	0.025 (5)	0.019 (5)

### Geometric parameters (Å, °)

**Table:**

Br1—C5	1.897 (6)	C8—H8	0.9800
N1—C1	1.260 (7)	C9—H9C	0.9600
N1—C2	1.429 (7)	C9—H9B	0.9600
C1—C1^i^	1.455 (11)	C9—H9A	0.9600
C1—H1	0.9300	C10—H10C	0.9600
C2—C7	1.393 (9)	C10—H10A	0.9600
C2—C3	1.403 (8)	C10—H10B	0.9600
C3—C4	1.380 (8)	C11—C12	1.483 (11)
C3—C8	1.513 (9)	C11—C13	1.533 (12)
C4—C5	1.374 (9)	C11—H11	0.9800
C4—H4	0.9300	C12—H12B	0.9600
C5—C6	1.379 (9)	C12—H12A	0.9600
C6—C7	1.393 (9)	C12—H12C	0.9600
C6—H6	0.9300	C13—H13A	0.9600
C7—C11	1.542 (9)	C13—H13B	0.9600
C8—C9	1.501 (12)	C13—H13C	0.9600
C8—C10	1.507 (10)		
			
C1—N1—C2	118.9 (5)	C8—C9—H9B	109.5
N1—C1—C1^i^	120.3 (7)	H9C—C9—H9B	109.5
N1—C1—H1	119.9	C8—C9—H9A	109.5
C1^i^—C1—H1	119.9	H9C—C9—H9A	109.5
C7—C2—C3	122.3 (5)	H9B—C9—H9A	109.5
C7—C2—N1	119.3 (5)	C8—C10—H10C	109.5
C3—C2—N1	118.4 (5)	C8—C10—H10A	109.5
C4—C3—C2	118.2 (5)	H10C—C10—H10A	109.5
C4—C3—C8	120.0 (5)	C8—C10—H10B	109.5
C2—C3—C8	121.8 (5)	H10C—C10—H10B	109.5
C5—C4—C3	119.9 (6)	H10A—C10—H10B	109.5
C5—C4—H4	120.1	C12—C11—C13	111.5 (8)
C3—C4—H4	120.1	C12—C11—C7	110.3 (6)
C4—C5—C6	121.9 (6)	C13—C11—C7	114.0 (6)
C4—C5—Br1	119.2 (5)	C12—C11—H11	106.9
C6—C5—Br1	118.9 (5)	C13—C11—H11	106.9
C5—C6—C7	119.9 (6)	C7—C11—H11	106.9
C5—C6—H6	120.1	C11—C12—H12B	109.5
C7—C6—H6	120.1	C11—C12—H12A	109.5
C6—C7—C2	117.7 (6)	H12B—C12—H12A	109.5
C6—C7—C11	120.2 (6)	C11—C12—H12C	109.5
C2—C7—C11	122.1 (6)	H12B—C12—H12C	109.5
C9—C8—C10	112.5 (8)	H12A—C12—H12C	109.5
C9—C8—C3	111.2 (6)	C11—C13—H13A	109.5
C10—C8—C3	113.0 (5)	C11—C13—H13B	109.5
C9—C8—H8	106.5	H13A—C13—H13B	109.5
C10—C8—H8	106.5	C11—C13—H13C	109.5
C3—C8—H8	106.5	H13A—C13—H13C	109.5
C8—C9—H9C	109.5	H13B—C13—H13C	109.5
			
C2—N1—C1—C1^i^	−179.3 (8)	C5—C6—C7—C11	−178.1 (6)
C1—N1—C2—C7	−90.5 (7)	C3—C2—C7—C6	−3.5 (9)
C1—N1—C2—C3	92.9 (7)	N1—C2—C7—C6	−179.9 (6)
C7—C2—C3—C4	3.3 (8)	C3—C2—C7—C11	174.9 (5)
N1—C2—C3—C4	179.8 (5)	N1—C2—C7—C11	−1.6 (8)
C7—C2—C3—C8	−177.7 (5)	C4—C3—C8—C9	82.5 (8)
N1—C2—C3—C8	−1.2 (8)	C2—C3—C8—C9	−96.4 (8)
C2—C3—C4—C5	0.1 (9)	C4—C3—C8—C10	−45.1 (9)
C8—C3—C4—C5	−178.9 (6)	C2—C3—C8—C10	135.9 (7)
C3—C4—C5—C6	−3.3 (10)	C6—C7—C11—C12	108.2 (9)
C3—C4—C5—Br1	177.6 (5)	C2—C7—C11—C12	−70.1 (9)
C4—C5—C6—C7	3.1 (10)	C6—C7—C11—C13	−18.1 (10)
Br1—C5—C6—C7	−177.8 (5)	C2—C7—C11—C13	163.6 (7)
C5—C6—C7—C2	0.3 (9)		

Symmetry codes: (i) −*x*+1, −*y*+1, −*z*+1.

### Hydrogen-bond geometry (Å, °)

**Table:**

*D*—H···*A*	*D*—H	H···*A*	*D*···*A*	*D*—H···*A*
C8—H8···N1	0.98	2.40	2.880 (9)	109
